# Selective head cooling in the acute phase of concussive injury: a neuroimaging study

**DOI:** 10.3389/fneur.2023.1272374

**Published:** 2023-10-27

**Authors:** Alexa E. Walter, Xiaoxiao Bai, James Wilkes, Thomas Neuberger, Wayne Sebastianelli, Semyon M. Slobounov

**Affiliations:** ^1^Department of Kinesiology, The Pennsylvania State University, University Park, PA, United States; ^2^Department of Neurology, University of Pennsylvania, Philadelphia, PA, United States; ^3^Social, Life, and Engineering Science Imaging Center, The Pennsylvania State University, University Park, PA, United States; ^4^Department of Kinesiology, The Pennsylvania State University, University Park, PA, United States; ^5^Department of Biomedical Engineering, and Social, Life, and Engineering Science Imaging Center, Huck Institutes of the Life Sciences, The Pennsylvania State University, University Park, PA, United States; ^6^Department of Athletic Medicine, The Pennsylvania State University, University Park, PA, United States; ^7^Department of Orthopaedics, Penn State Health, State College, PA, United States; ^8^Department of Kinesiology, The Pennsylvania State University, University Park, PA, United States

**Keywords:** concussion, athletes, selective head cooling, magnetic resonance imaging, arterial spin labeling, magnetic resonance spectroscopy

## Abstract

**Introduction:**

Neurovascular decoupling is a common consequence after brain injuries like sports-related concussion. Failure to appropriately match cerebral blood flow (CBF) with increases in metabolic demands of the brain can lead to alterations in neurological function and symptom presentation. Therapeutic hypothermia has been used in medicine for neuroprotection and has been shown to improve outcome. This study aimed to examine the real time effect of selective head cooling on healthy controls and concussed athletes via magnetic resonance spectroscopy (MRS) and arterial spin labeling (ASL) measures.

**Methods:**

24 participants (12 controls; 12 concussed) underwent study procedures including the Post-Concussion Symptom Severity (PCSS) Rating Form and an MRI cooling protocol (pre-cooling (T1 MPRAGE, ASL, single volume spectroscopy (SVS)); during cooling (ASL, SVS)).

**Results:**

Results showed general decreases in brain temperature as a function of time for both groups. Repeated measures ANOVA showed a significant main effect of time (*F* = 7.94, *p* < 0.001) and group (*F* = 22.21, *p* < 0.001) on temperature, but no significant interaction of group and time (*F* = 1.36, *p* = 0.237). CBF assessed via ASL was non-significantly lower in concussed individuals at pre-cooling and generalized linear mixed model analyses demonstrated a significant main effect of time for the occipital left ROI (*F* = 11.29, *p* = 0.002) and occipital right ROI (*F* = 13.39, *p* = 0.001). There was no relationship between any MRI metric and PCSS symptom burden.

**Discussion:**

These findings suggest the feasibility of MRS thermometry to monitor alterations of brain temperature in concussed athletes and that metabolic responses in response to cooling after concussion may differ from controls.

## 1. Introduction

Sports-related concussion has become a growing health concern over the last decade as it is complex in terms of both symptomology and physiology of injury. While the physiology of concussion has been previously described and involves many biological processes, one of the major theoretical constructs surrounding alterations in brain biochemistry and its translation to clinical symptom presentation includes neurovascular decoupling (1).

Cerebral blood flow (CBF) is coupled to neuronal activity and cerebral glucose metabolism. Within normal levels of CBF, the brain is able to extract 50% of the oxygen and 10% of the available glucose from arterial blood, however following concussive injury, CBF can be reduced up to 50% of its normal values (2, 3). Increased energy demand, and therefore an increase in glucose consumption and a reduction in cerebral blood flow, results in de-coupling between energy supply and demand – the cellular energy crisis (1). Due to post-injury decreases in CBF, failure to appropriately match CBF with surges in metabolic demands of the brain can lead to temporary or permanent alterations in neurological functioning and symptom presentation (4).

Induced hypothermia following traumatic brain injury (TBI) has become a common treatment protocol and many major medical societies recommend this procedure as standard of care therapy (5); however, there is debate regarding its utility and effect on outcomes (6, 7). Hypothermia is thought to have biologically-relevant neuroprotective effects and, when employed acutely, can decrease endogenous antioxidant consumption and lipid peroxidation (8), attenuate cell death (9), increase autophagy (9), reduce intracranial pressure (10), reduce inflammation (11), and reduce synaptic transmission efficacy (12). Additionally, in the sub-acute and chronic phases of injury, hypothermia has been shown to attenuate blood brain barrier disruption, reduce cerebral edema, inhibit inflammation, attenuate microcirculatory dysfunction and stimulate neurogenesis, gliogenesis, angiogenesis, and synaptogenesis [for a review see (10)]. It has also been previously linked to positive patient outcomes (13–16). However, the use of mild hypothermia with TBI has been met with some controversy, especially its role as an early neuroprotectant. Animal models have shown benefits (17), but its translation into human studies has not been clearly demonstrated (18–20).

Selective head cooling has been previously studied (21–24) and typically involves mild therapeutic hypothermia applied in simple, non-invasive ways. Using advanced imaging techniques, like Magnetic Resonance Spectroscopy (MRS), changes in temperature can be measured through estimation from the chemical shift difference observed between water and N-acetylaspartate (NAA) peaks at each voxel (25). MRS studies have shown that head cooling produced significant brain temperature reductions of 0.458° C after only 30 min (21) and decreases of 1.7°C after 1 h (25).

This study specifically utilized the WElkins sideline cooling system. The first version of this cooling helmet showed a mean brain temperature decrease of 1.6°C while core temperature did not drop below 37°C until 4–5 h after cooling began (24). On average, a 1.84°C reduction in brain temperature was seen within 1 h of using this helmet and it took 6.67 h before hypothermia (34°C) was reached (26). Additionally, previous work from our group using this device in concussed individuals showed nonsignificant changes in the default mode network using resting state-functional MRI (rs-fMRI) and significant increases in CBF using arterial spin labeling (ASL) after 30 min of cooling (27). However, this study was limited by design, as the individual was removed between scans for application of the cooling device. The time lags surrounding the cooling administration protocol, as well as the lack of direct measure of temperature via MRI-based measures, like MRS, prevented real time observation of temperature changes in the brain.

Therefore, this follow-up study aimed to examine real time effect of selective head cooling on control and concussed athletes using ASL and MRS sequences. Our specific hypothesis was that selective head cooling in acute phase of injury would result in changes to the neurovascular state of the brain after concussion. Accordingly, we hypothesized that (1) selective head cooling would cause decreases in temperature in both control and concussed individuals as assessed by MRS; and (2) blood flow, as assessed by ASL, would be lower in concussed individuals at baseline (pre-cooling) and would increase during the cooling protocol, as compared to controls.

## 2. Methods

### 2.1. Participants

Participants were recruited from Pennsylvania State University intercollegiate athletics. Concussed individuals were diagnosed by Penn State Sports Medicine physicians and study procedures were completed within the acute phase (<10 days) of injury. Exclusion criteria included (a) under 18 years of age; (b) any psychiatric or developmental disorder diagnoses; (c) use of antidepressants, anxiolytics, anticonvulsants, antipsychotics, hypnotics, stimulants, or antihistamines; (d) alcohol or substance abuse; (e) previous spinal cord injury; (f) contraindication to MRI; and (g) previous history of concussion (for controls only).

Pre-study sample size calculations were done using previously published findings (27) and a sample size of 12 per group was indicated as being large enough to detect appropriate power. 27 athletes were screened and *n* = 3 were ineligible for inclusion in the study (*n* = 1 development disorder diagnosis and *n* = 2 contraindication to MRI). The remaining 24 athletes were enrolled in the study. This study followed ethical guidelines approved by the Institutional Review Board at the Pennsylvania State University. All participants provided written informed consent before participating in the study.

### 2.2. Procedures

All participants completed a demographic information form and the Post-Concussion Symptom Severity (PCSS) Rating Form at the time of their scanning session. This form rates 22 different symptoms on a scale of 0 (none) to 6 (severe). All participants recorded how they were feeling that day, and concussed participants also recorded symptoms within 24 h after injury.

**Figure 1:**
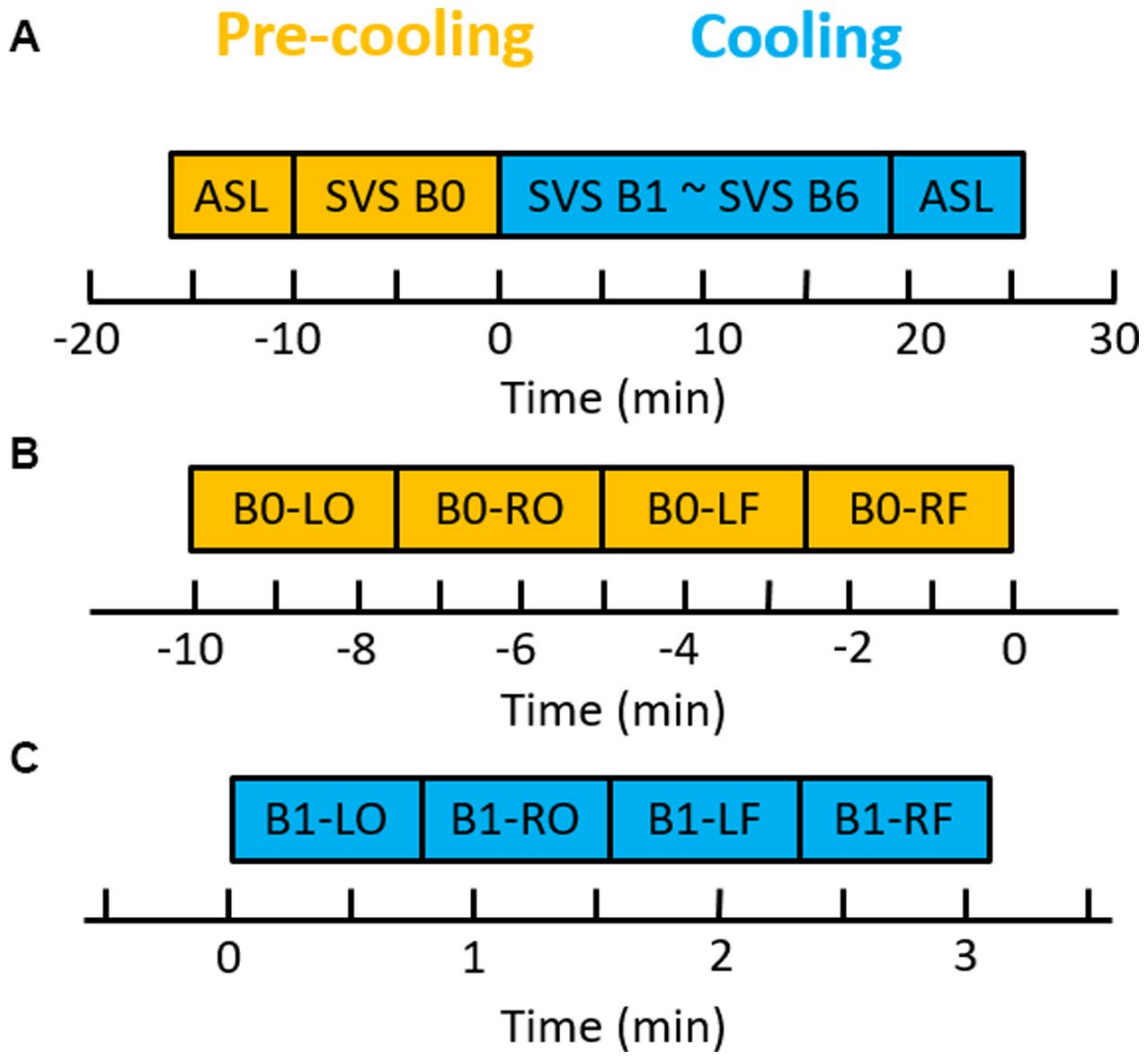
Brain cooling scan paradigm. **(A)** Diagram of ASL and SVS paradigm. One ASL and one block of SVS (SVS B0) were acquired before the brain cooling system was turned on. Six blocks of SVS and ASL were acquired during the brain cooling system run. **(B)** One block of SVS before brain cooling. **(C)** One of six blocks of SVS during the brain cooling. LO, left occipital; RO, right occipital; LF, left frontal; and RF, right frontal. B0, the block 0 of SVS.

All participants then underwent MRI scans with a cooling procedure (MRI experimental design outlined in Figure 1; cooling procedure approximately 25 min). This study was adapted from a pilot study using the same cooling device (27). Four regions of interest (right occipital, left occipital, right frontal, left frontal) were selected as focal points for analyses given their relevance and high sensitivity to concussive injury (28–33). After completing the MRI scans, all participants again completed the PCSS rating form (34).

### 2.3. MRI acquisition

All imaging was done on a 3 tesla Magnetom Prisma-Fit scanner (Siemens Medical Systems, Erlangen, Germany) with a 20-channel head coil. Each scanning session (Figure 1A) consisted of:

1. 3D T1-weighted MPRAGE: TR, 2,200 ms; TE, 2.45 ms; TI, 900 ms; FOV, 256 × 256 mm^2^; number of slices, 192; slice thickness, 1.0 mm; 1.0 mm isotropic resolution; flip angle, 8°; scan time, 5 min 38 s.
2. 3D PCASL (Siemens advanced 3D ASL package), TI, 3,600 ms; Bolus Duration, 1,800 ms; TR, 4,000 ms; TE, 14.62 ms; field of view 192 × 192 mm^2^, matrix 64 × 64, slice thickness 3 mm, number of slices, 42; 1.0 mm isotropic resolution; Perfusion model, PICORE Q2T; Suppression model, GRAY-White-Strong; M0 TR 5,000 ms; bandwidth 2,694 Hz/pixel; labeling and control pairs, 8; scan time, 4 min 42 s.
3. Single volume spectroscopy SVS (PRESS); TR, 3,000 ms; TE, 145 ms; volume, 20 × 20 × 20 mm^3^; acquisition duration, 1,024 ms; bandwidth, 1,000 Hz; data points, 1,024; the number of averages, 8; prep scan image number, 4; coil combination time, 10 s; water suppression method, variable power radio frequency pulses with optimized relaxation delays (VAPOR); total scan time, 46 s.

### 2.4. Image acquisition

An *auto align pulse sequence* (AAscout) was used to align the acquisition slices of the T1 MPRAGE and PCASL scans parallel to the anterior and posterior commissure (*AC*–*PC*) *plane* and centered on the brain. The single volume of SVS was manually placed by using the axial T1 MPRAGE for planning. After obtaining 3D T1 MPRAGE, pre-cooling PCASL and one block of PRESS SVS, 6 blocks of PRESS SVS and cooling PCASL (Figure 1A) were acquired immediately after the brain cooling system was started. Each block of PRESS SVS composed of four SVS scans located in the white matter close to the left occipital, right occipital, and left frontal and right frontal regions, respectively. The total acquisition times for pre-cooling SVS block (Figure 1B) and for each cooling SVS block (Figure 1C) were 9 min 4 s and 3 min 4 s, respectively. Automatic shimming was performed only for the pre-cooling SVS scan using the Siemens “Advanced” shimming option and took around 90 s. The water frequency position was obtained from the residual water peak of the acquisition spectrum. The variable power radio frequency pulses with optimized relaxation delays (VAPOR) was used for the water suppression (35). Full details regarding the MRS protocol can be found in Supplementary File 1 as outlined by the reporting standards by Lin et al. (36).

### 2.5. Image processing

#### 2.5.1. Arterial spin labeling

**Figure 2:**
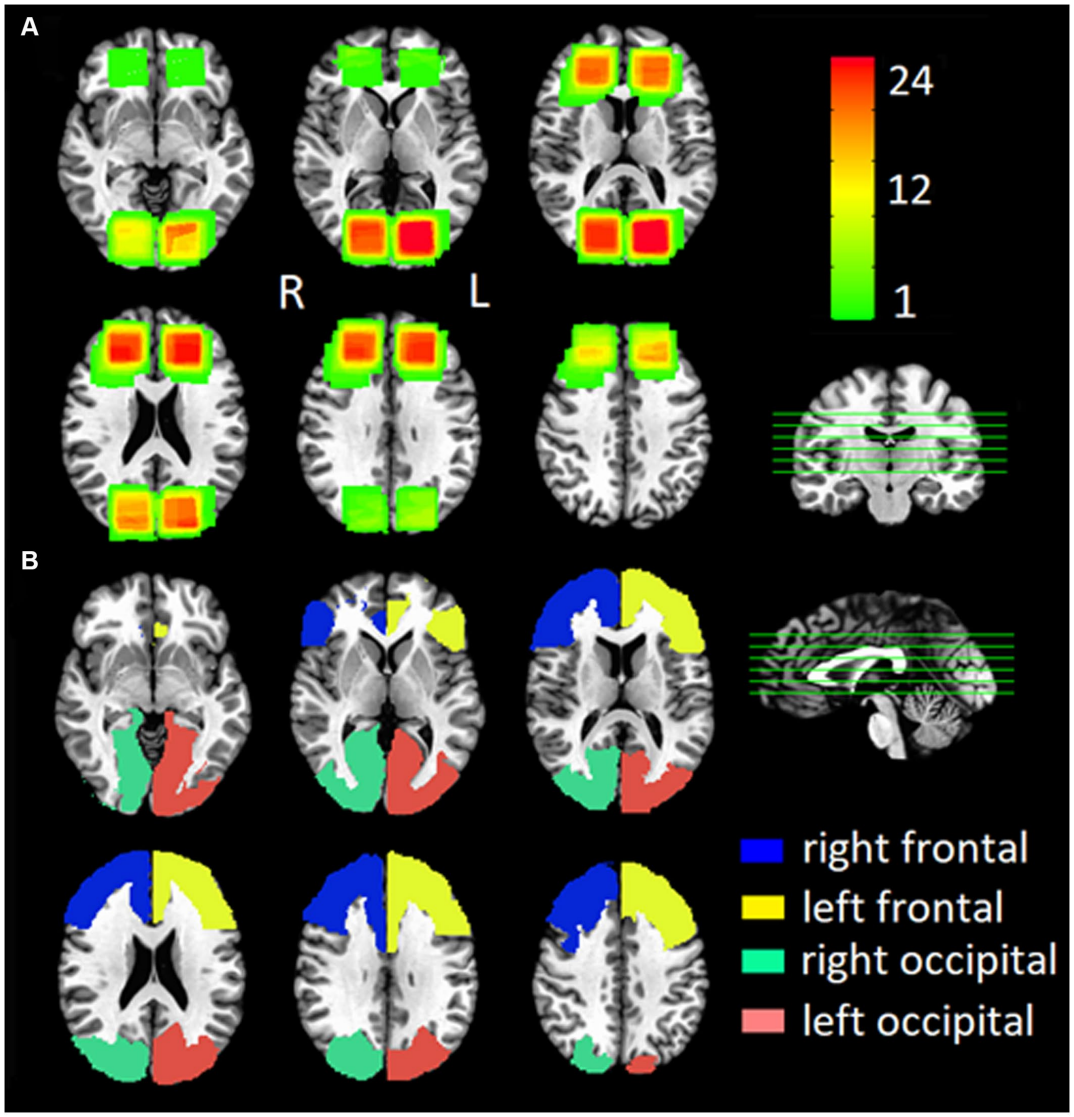
Group Locations of **(A)** the single voxel spectroscopy (SVS) location for all participants and **(B)** the anatomical location of the ROI for the ASL sequence for all participants.

The regional cerebral blood flow (reCBF) map was automatically calculated for each PCASL acquisition by using the Siemens advanced 3D ASL package. To calculate the reCBF in the Siemens advanced 3D ASL package for the single TI PCASL method the approach of Wang et al. (37) was used:


(1) $$f=\frac{\lambda \Delta MR_{1a}}{2\alpha M_{0}\left[\exp\left(-wR_{1a}\right)-\exp\left(-\left(\tau +w\right)R_{1a}\right)\right]}$$

where *f* is the reCBF in mL/100 g/min; *λ* is 0.9 mL/g blood/tissue water partition coefficient, *α* is 60% inversion efficiency using suppression model GRAY-White-Strong (38). *M*_0_ is fully relaxed image intensity, Δ*M* is signal difference (control/labeled), *τ* is 1,800 ms duration of labeling pulse, *w* is the post-labeling delay *w* = TI-*τ*, TI is 3,600 ms inversion time, Inversion Time TI1/TI2 are 700/1,990 ms inversion times, TI2 = TI1+ w (transit time), and *R*_1*a*_ is 0.606 s^−1^ at 3 T longitudinal relaxation time of blood. To create the ROIs, the reCBF was evaluated in predefined regions-of-interest (ROIs) for control and concussion groups. One volume of interest was constructed from the group overlapping of two PCASL images of patients and control (Figure 2). Four gross anatomical ROIs were constructed from the standard reference template (AFNI: TT_N27_EZ_ML TLRC atlas) (39) as follow (Figure 2): left and right frontal (Inferior_Triangularis_Fronal Gyrus + Middle_Frontal_Gyrus + Superior_Frontal_Gyrus + Anterior_Cingulate_Cortex + Superior_Medial_Gyrus); left and right occipital (Calcarine_Gyrus + Cuneus + Lingual_Gyrus + Superior_Occipoital_Gyrus + Middle_Occipital_Gyrus).

The relCBF maps were preprocessed and analyzed by using AFNI (Analysis of Functional NeuroImages, http://afni.nimh.nih.gov/). For each subject, preprocessing procedures included co-registration to the T1-weighted anatomical image, co-registration between pre-cooling and cooling 3D PCASL images and spatial normalization to standard space (TT_N27 + MNI) using a 12-parameter affine registration.

#### 2.5.2. Single voxel spectroscopy

For each SVS scan, the single-voxel spectra was processed using the time-domain fitting routine AMARES algorithm in the jMRUI 6.0 beta software package with water removal (HLSVD) turned off and apodization off (jMRUI, Java Magnetic Resonance User Interface) (35, 40–42). AMARES fitting of the water and NAA peaks, representative spectra, and SNR and linewidth information is presented in Supplementary File 2.

The temperature was calculated according to the following equation (41, 43),


(2) $$T=37{}^{\circ}C+100{}^{\circ}C/\text{ppm}\times \left(4.7\text{ppm}-\delta_{H_{2}O}+\delta_{NAA}-2.035\text{ppm}\right)$$

Where *T* is brain temperature in degree Celsius (°C), and $\delta_{H_{2}O}$ and $\delta_{NAA}$ are the center of the water and N-acetylaspartate NAA fitted peaks in ppm in the acquired spectrum. For SVS, each single-voxel of each subject was automatically co-registered to the individual T1-weight anatomical image by using the Gannet 3.0 algorithm (GannetCoRegister) (44). The registered mask maps were then spatially normalized to the standard space (TT_N27 + trlc) using the 12-parameter affine registration (Figure 2).

### 2.6. Welkins sideline cooling system

The cooling system used in this study was the WElkins Sideline Cooling System (Spartan Medical Inc., Silver Spring, MD). This cooling system circulates temperature-controlled coolant (10°C/50°F; containing water, propylene glycol, disinfectant, and surfactant) through umbilical tubing to the MR compatible cooling headliner which is fitted to the participants’ head before they are set in the MRI scanning position (Supplemental File 3).

### 2.7. Phantom scan

To test the brain cooling system, single volume spectroscopy (SVS; PRESS) (42, 45) was applied on a standard Siemens MRS phantom (Model# 7576577) comprised of a 170 mm diameter sphere containing distilled water and 8.2 g Na C_2_H_3_O_2_ and 9.6 g C_3_H_5_O_3_Li per liter of water. The 20 × 20 mm voxel was placed well within the sphere about 2.5 cm away from the cooling head cap. After placing the voxel, 36 SVS experiments were acquired using the same parameters as described above. After 18 SVS scans, the braining cooling system was turned on (Supplemental File 4).

### 2.8. Statistical analysis

All statistical analysis was done using SPSS V26 and significance was set *a priori* at *p* < 0.05. Examination of variable distribution for normality was conducted prior to statistical analysis to identify whether the data met the required assumptions. Symptom rating as assessed by PCSS was examined with a repeated measures ANOVA with main effects of group and time and the interaction of group and time. Changes in temperature, as calculated from SVS, from the pre-cooling block were calculated for each during cooling SVS block. A repeated measures ANOVA, with main effects of group and time and an interaction term of group by time, was run to assess temperature. To assess changes in cerebral blood flow, a generalized linear mixed model was run with time as a fixed effect and subject as a random effect. Sex was used as a covariate and appropriate interactions were included. For significant interactions, a simple effects analysis using sequential Bonferroni correction was used to identify location of differences. Spearman’s correlations were run to examine the relationship between MRI metrics (ASL whole brain and individual ROIs) and symptom burden from PCSS.

## 3. Results

### 3.1. Participant demographics and symptom reporting

A total of 24 participants were enrolled (12 concussion and 12 control) and demographic information is presented in Table 1. Both groups had a mean age around 20 years old. The concussion group came from a variety of Division I sports and were scanned at a mean 6.58 days post-injury. They reported a mean of 35.82 symptoms on PCSS within the first 24 h of their injury. To examine the change in PCSS from pre-cooling to post-cooling procedures, a repeated measures ANOVA was run and showed no significant effect of group (*F* = 0.99, *p* = 0.33), time (*F* = 1.38, *p* = 0.25), or their interaction (*F* = 3.11, *p* = 0.09).

**Table 1:** Participant demographic information and total symptom rating at various timepoints.

	Control (*n* = 12)	Concussion (*n* = 12)
Sex^a^ *(n; %)*	Male (6; 50); Female (6; 50)	Male (8; 66.7); Female (4; 33.3)
Age^b^ *mean ± std dev*	19.58 *±* 0.26 years	19.75 *±* 0.33 years
Sport *(n; %)*	Track and Field (11; 91.7)	Softball (1; 8.3)
		Soccer (1; 8.3)
		Football (2; 16.7)
		Dance (1; 8.3)
	Swimming (1; 8.3)	Rugby (4; 33.3)
		Track & Field (1; 8.3)
		Wrestling (1; 8.3)
		Tennis (1; 8.3)
Years playing the sport^b^ *mean ± std dev*	7.58 *±* 0.51	10.00 *±* 1.56
Number of previous concussions *(n; %)*	0 (12; 100)	1 (8; 66.7)
		2 (4; 33.3)
Days since injury at scanning session *mean ± std dev (range)*	NA	6.58 *±* 0.61 (2–10)
Average symptom rating at time of injury *mean ± std dev (range)*	NA	35.82 *±* 7.99 (1–92)
Average symptom rating before scanning *mean ± std dev (range)*	3.17 *±* 1.61 (0–19)	9.83 *±* 5.04 (0–59)
Average symptom rating after scanning *mean ± std dev (range)*	4.73 *±* 1.37 (0–16)	5.67 *±* 3.05 (0–28)

^a^Nonsignificant by Chi square. ^b^No difference between groups by independent *t*-test.

### 3.2. SVS results

**Figure 3:**
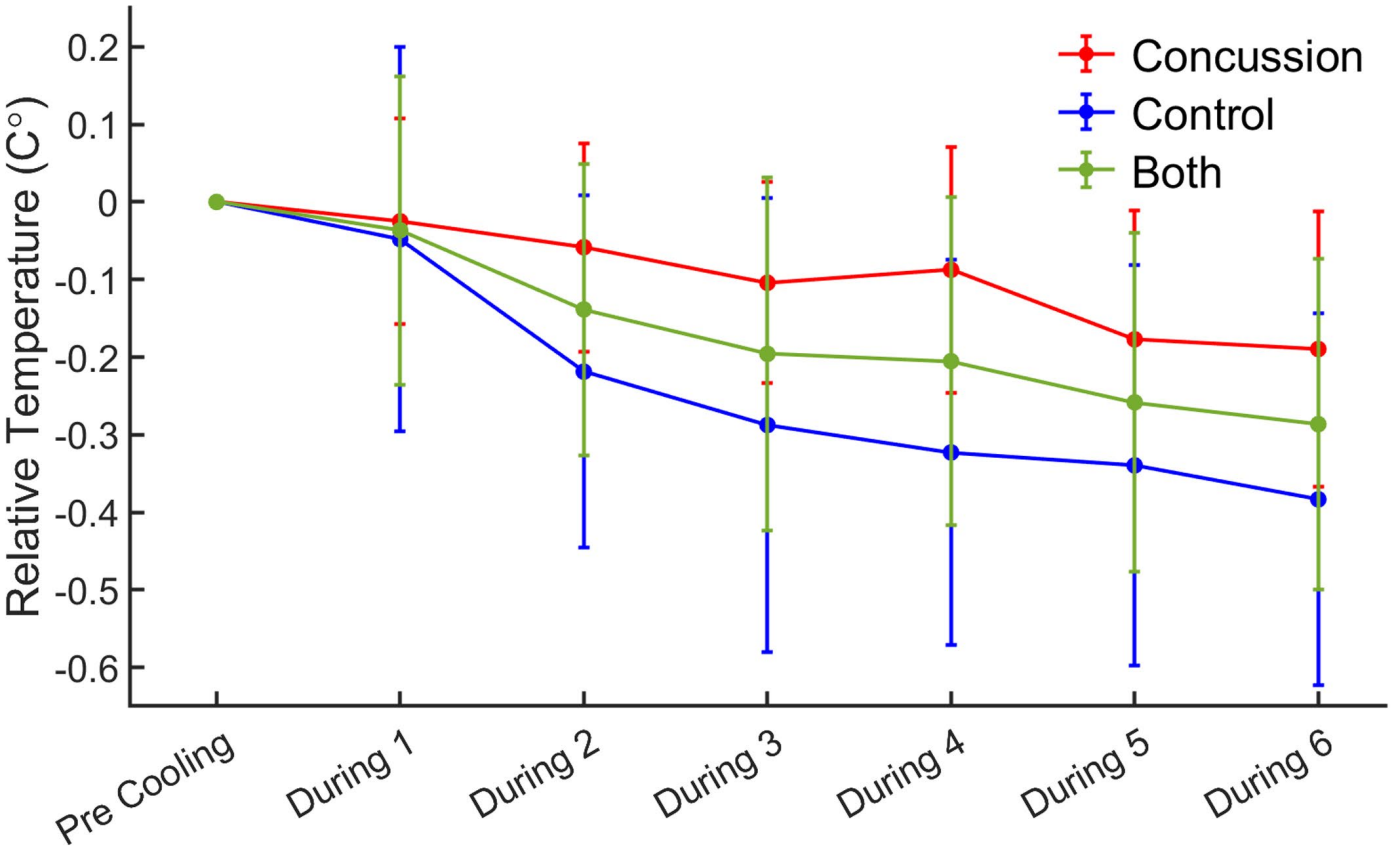
Relative change in temperature from pre-cooling to during cooling blocks by group. *Red = concussion; blue = control; green = whole group sample*. For the concussed group, simple main effects analysis showed that SVS block 5 (*p* = 0.023) and 6 (*p* = 0.015) were significantly different from the pre-cooling SVS block. For the control group, simple main effects analysis showed that SVS blocks 2 (*p* = 0.005), 3 (*p* < 0.001), 4 (*p* < 0.001), 5 (*p* < 0.001), and 6 (*p* < 0.001) were all significantly different from the pre-cooling SVS block.

Changes in temperature during each cooling block, as measured by single volume spectroscopy (SVS), were calculated as the change in temperature from the pre-cooling block (Figure 3). A repeated measures ANOVA demonstrated a significant main effect of time (*F* = 7.94, *p* < 0.001) and group (*F* = 22.21, *p* < 0.001). The interaction term of group by time was non-significant (*F* = 1.36, *p* = 0.237). Simple main effects analysis showed that for the concussed group, only SVS block 5 (*p* = 0.023) and 6 (*p* = 0.015) were significantly different from the pre-cooling SVS block. For the control group, blocks 2 (*p* = 0.005), 3 (*p* < 0.001), 4 (*p* < 0.001), 5 (*p* < 0.001), and 6 (*p* < 0.001) were all significantly different from the pre-cooling SVS block.

### 3.3. ASL results

**Figure 4:**
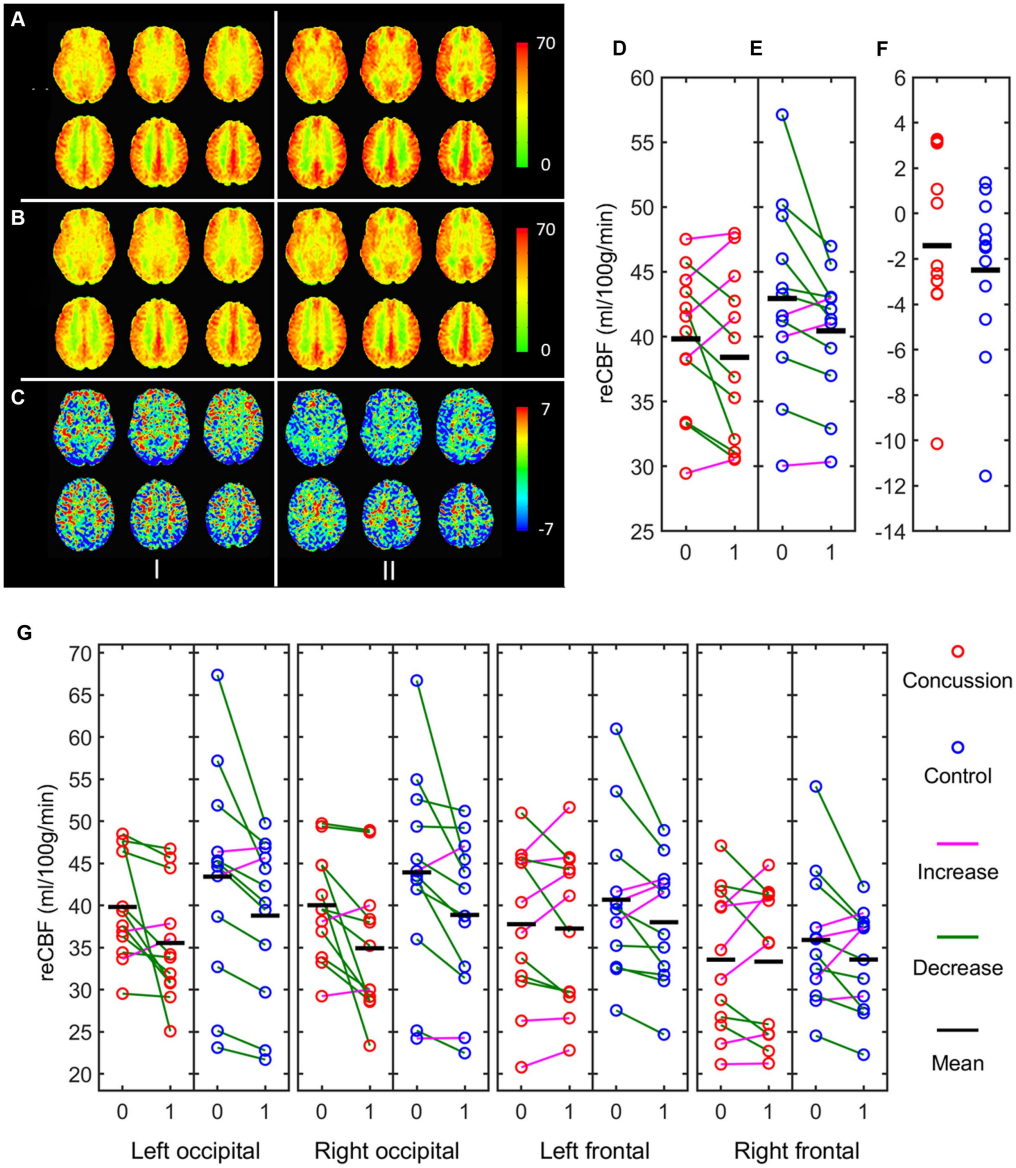
Mean ASL maps of concussed (I) and control (II) participants. **(A)** pre-cooling; **(B)** during cooling; **(C)** Difference of during—pre-cooling; **(D)** Whole brain mean ASL of concussed, **(E)** controls, and **(F)** difference of during—pre-cooling using the entire brain ROI; **(G)** difference of during—pre-cooling using each individual ROI *red = concussion; blue = control; 0 = pre-cooling; 1 = during-cooling.*

Changes in cerebral blood flow (CBF), as measured by arterial spin labeling (ASL), are presented in Figure 4 for whole brain CBF and for each individual ROI. Generalized linear mixed model analyses were run for each ROI and the fixed effects summary is presented in Table 2. The occipital left ROI revealed only a significant main effect of time [t = −3.361, *p* = 0.002, 95% CI: (−6.390, −1.595)] whereas pre-cooling (42.94 ± 1.75) had higher cerebral blood flow values than during cooling (38.95 ± 1.60). The occipital right ROI also revealed a significant main effect of time [t = −3.659, *p* = 0.001, 95% CI: (−6.432, −1.859)] whereas pre-cooling (42.38 ± 1.52) had higher cerebral blood flow values than during cooling (38.24 ± 1.29). Analyses for the frontal left and frontal right ROI revealed no significant main effects for time, group, or sex (*p* > 0.05).

**Table 2:** Fixed effects for each ROI for cerebral blood flow.

	Occipital left		Occipital right		Frontal left		Frontal right	
Model term	F	β (95% CI)	F	β (95% CI)	F	β (95% CI)	F	β (95% CI)
Group	0.42	−5.98 (−14.65, 2.70)	1.82	−6.41 (−13.79, 0.97)	0.03	−7.05 (−16.60, 2.50)	−0.001	−6.17 (−14.63, 2.28)
Time	11.29	**−3.94 (−7.33, −0.55)**	13.39	**−3.84 (−7.01, −0.60)**	3.24	−2.67 (−5.20, −0.14)	2.01	−2.32 (−4.90, 0.25)
Sex	0.03	−4.50 (−13.09, 4.09)	1.23	−6.03 (−12.99, 0.95)	0.26	−3.55 (−13.12, 6.03)	0.43	−2.99 (−11.52, 5.55)
Group * time	0.002	−0.10 (−4.90, 4.69)	0.08	−0.62 (−5.19, 3.95)	1.47	2.15 (−1.43, 5.73)	1.34	2.09 (−1.55, 5.73)
Group * sex	1.66	7.98 (−4.54, 20.50)	1.66	6.48 (−3.69, 16.64)	2.37	10.64 (−3.31, 24.60)	2.65	10.03 (−2.41, 22.46)

Bolded: significant *p* < 0.05. CI, confidence interval.

### 3.4. Correlation with symptom burden

Spearman’s correlations revealed no significant relationship (*p* > 0.05) between any MRI metric (ASL whole brain and each individual ROI) and PCSS symptom burden pre-cooling, post-cooling, or the change in symptom reporting from pre-to post-cooling.

## 4. Discussion

This study aimed to follow up on previous work (27) to determine if selective head cooling affected the physiology of the brain and clinical symptom presentation during the acute phase of concussion. Using ASL and MRS sequences, we were able to directly examine the effect of the cooling device on imaging metrics in real time.

There are several findings of interest that will be discussed. Firstly, the cooling device used in this study was shown to lower brain temperature as assessed via SVS measurements. Both in phantom data and human data, brain temperature was reduced within 30 min of selective head cooling. This confirms results from other studies using this device (24, 26) and the non-invasive methodology used makes its future implementation potentially more feasible.

In both groups, there were mostly minor decreases in temperature during the cooling procedure, however, the control group had a quicker and more pronounced decrease compared to the concussion group. Given the neurometabolic cascade that occurs after concussion (1), there may be biological rationale for why the concussion group had a slower reaction to temperature. One theory is that vascular response mechanisms in an injured brain may be different and delayed compared to the non-injured control brain. Deficits in neurovascular reactivity, like decreases in CBF, may cause the injured brain to take longer to respond to perturbations in temperature. Another theory is that neuroinflammation could be interfering with the process. Hypothermia is thought to help inhibit inflammation through microglia (46, 47) however, given that this cohort is days post-injury, the inflammation process related to brain injury may affect the expected processes.

In regard to cerebral blood flow changes as assessed by ASL, pre-cooling there were non-significant regional differences between control and concussed participants with concussed individuals showing lower CBF measures. A review (48) found that most studies done in mild TBI demonstrate decreases in CBF relative to controls suggesting that there is failure to match CBF with metabolic demands of the brain after concussion. However, there was great heterogeneity of findings with some studies reporting decreases, increases, or no change in CBF relative to control populations. In the college athletic population in particular, studies have primarily reported no group differences (49–51) or decreases in CBF relative to controls (26, 27, 52–55) across various timepoints post-injury. Results could be confounded by different timepoints since injury, ASL sequence parameters, imaging and statistical analysis techniques among other injury factors. It should be noted that cardiovascular fitness of athletes participating in different sporting activities may influence vascular reactivity and could be examined as a potential confounder on CBF in future studies.

During the cooling procedure, there was a significant effect of time on CBF for both groups in the occipital lobe ROIs. The frontal lobe ROIs however, showed less pronounced decreases in CBF during cooling procedures. This is in contrast to our previous work that examined CBF changes after the cooling procedure was finished and found a significant increase in CBF in concussed individuals compared to controls. These differences in CBF may reflect different physiological mechanisms of the brain in response to thermoregulatory challenge. Changes in cortical temperature are thought to regulate regional CBF through autoregulation of the microcirculation (56) and this autonomic response is a key homeostatic mechanism. Autonomic dysfunction after concussion is considered a major factor in symptomology post-injury (57). Given the role of the autonomic system in cerebral perfusion, this could be contributing to both the differential reactions seen in cerebral blood flow measures and the wide range of symptomology reported after concussion.

When examining the relationship of clinical symptom reporting, = there was no significant effect of group (control vs. concussion), time (pre- vs. post-cooling), or their interaction on PCSS. This suggests that the device does not exacerbate self-reported symptomology. Even though there was a non-significant effect of time, it is interesting to note that the concussed group did report a decrease in symptoms from pre-to post-cooling procedures.

This should be interpreted with caution as symptom reporting is a relatively crude outcome measure that may be influenced by other external factors and may change daily. Future work should expand the outcome measures collected to include additional tests across time and across modality as there is some evidence from other groups that head and neck cooling procedures after concussion diagnosis (within minutes) led to shorter return-to-play times than those who received standard care (58). The relationship between these more crude clinical factors and the underlying pathobiology is still unknown and should be more rigorously explored.

While overall main effects and interactions including sex were non-significant for ASL, there were interesting sex differences in some simple post-hoc contrasts analyses. The group by sex interaction showed a differential pattern of response to the cooling procedures with males having decreased CBF at each ROI and females having stable or increased CBF at each ROI during cooling. While not statistically significant, these findings may be important for future work given other work that has highlighted sex differences in different imaging modalities including resting state fMRI (59), diffusion tensor imaging (60), and ASL (54, 60). Future studies in larger cohorts should consider these potential sex differences.

This study is not without limitations. This was done in a fairly small sample size and homogenous population, limiting generalizability of the results. The group was also heterogeneous in regard to the sport they participated in which may influence concussion injury factors like location of impact or injury severity. Furthermore, the cooling period was relativity short (around 25 min) which may not be the optimal amount of time to see the complete response of CBF to temperature changes. This is complicated by the fact that these individuals were still in the acute period of their injury, so lengthy times in the scanner and symptom exacerbation were critical to avoid as well. Additionally, most of the females in this study had long hair, as compared to the males who tended to have shorter hair. This could complicate findings as the cooling helmet may have been more effectively able to deliver the coolant in this time frame to short-haired individuals. In regard to the MRS procedures, the equation used to calculate temperature is from a previous study with a small sample size and there is no consensus on which equation is most accurate in head temperature estimations. We also did not have the ability to measure temperature internally during the phantom procedures which could influence results. Lastly, the placement of the voxels during the protocol was not randomized which also may affect temperature results.

## 5. Conclusion

This study utilized magnetic resonance imaging to examine the real time effects of selective head cooling in concussed individuals. Overall findings revealed that this cooling device does decrease brain temperature and cerebral blood flow decreases in response to cooling procedures. The clinical utility of selective head cooling is still to be determined and upon further validation, could be a useful tool in clinical practice of concussion during acute phase of injury.

## Data availability statement

The raw data supporting the conclusions of this article will be made available by the authors, without undue reservation.

## Ethics statement

The studies involving humans were approved by the Pennsylvania State University Institutional Review Board. The studies were conducted in accordance with the local legislation and institutional requirements. The participants provided their written informed consent to participate in this study.

## Author contributions

AW: Conceptualization, Data curation, Formal analysis, Writing – original draft. XB: Conceptualization, Formal analysis, Methodology, Writing – original draft. JW: Data curation, Project administration, Writing – review & editing. TN: Conceptualization, Methodology, Writing – review & editing. WS: Resources, Writing – review & editing. SS: Conceptualization, Resources, Supervision, Writing – original draft.
