# Supplementary material for: Selective head cooling in the acute phase of concussive injury: a neuroimaging study
Source: Front Neurol. 2023 Oct 27;14:1272374. doi: 10.3389/fneur.2023.1272374 (PMC10641407; doi:10.3389/fneur.2023.1272374)
Supplement: Supplementary file 1 [file Table_1.DOCX]

**Supplemental File 1.**

**MRS Protocol Checklist**

| Site: Penn State University, University Park, PA, USA | |
| --- | --- |
| 1. Hardware |  |
| 1. Field strength (T) 2. Manufacturer 3. Model 4. RF coil 5. Additional hardware | 1. 3T 2. Siemens 3. Prisma Fit 4. 20 channel head coil 5. NA |
| 1. Acquisition |  |
| 1. Pulse sequence 2. VOI 3. VOI size 4. TR, TE 5. Number of acquisitions 6. Additional parameters 7. Water suppression method 8. Shimming method | 1. SVS PRESS 2. Left and right occipital, left and right frontal 3. 20 mm^3^ 4. TR 3000ms; TE 145ms 5. 8 averages per 6. 1024 data points, bandwidth 1000Hz 7. VAPOR 8. Vendor-supplied auto-shimming |
| 1. Data Analysis Methods and Outputs |  |
| 1. Analysis Software 2. Output measure | 1. jMRUI 6.0 2. Temperature (°C) |
| 1. Data Quality |  |
| 1. Reported variables 2. Data exclusion criteria 3. Quality measures 4. Sample spectrum | 1. SNR, linewidth 2. No subjects excluded 3. SNR, linewidth 4. See Supplementary File 2 |
