# Supplementary material for: Selective head cooling in the acute phase of concussive injury: a neuroimaging study
Source: Front Neurol. 2023 Oct 27;14:1272374. doi: 10.3389/fneur.2023.1272374 (PMC10641407; doi:10.3389/fneur.2023.1272374)
Supplement: Supplementary file 2 [file Table_2.DOCX]

**Supplementary File 2.**

**Figure S2:** example of AMARES fitting and temperature estimation. A) AMARES fitting. Peak 1 is the H2O peak, Peak 2 is NAA peak, Peak 3 is Cr and peak 2 is the Cho peak. B) Temperature estimation based on the Freq.(ppm) of two peaks.

A)

| Experiment | | | | Algorithm parameters | | | |
| --- | --- | --- | --- | --- | --- | --- | --- |
| Date | |  | | Algorithm used | | AMARES | |
| Directory | |  | | Iterations | | 12 | |
| Current file | |  | | Points Signal/Quant. | | 1024/1024 | |
| Calc. time (ms) | | 85 | | Truncated Points | | 0 | |
| Sampling Int. (ms) | | 1.000 | | Asked/found | | 4 / 4 | |
| Zero Order (deg) | | 4.5 +/- 0.0 | | Residue St.D | | 3.60E1 | |
| Begin Time (ms) | | -0.04 +/- 0.00 | | S/N | | 89.71 | |
| Name | Freq.(ppm) | | Linew.(Hz) | Amplitude | Sd. Amp. | | Phase(deg) |
| 1-L | -0.022 | | 9.28 | 3.018E3 | 12.10 | | 0.0 |
| 2-L | -2.676 | | 7.88 | 650.60 | 11.15 | | 0.0 |
| 3-L | -1.657 | | 7.73 | 275.71 | 12.12 | | 0.0 |
| 4-L | -1.467 | | 8.91 | 215.80 | 13.22 | | 0.0 |


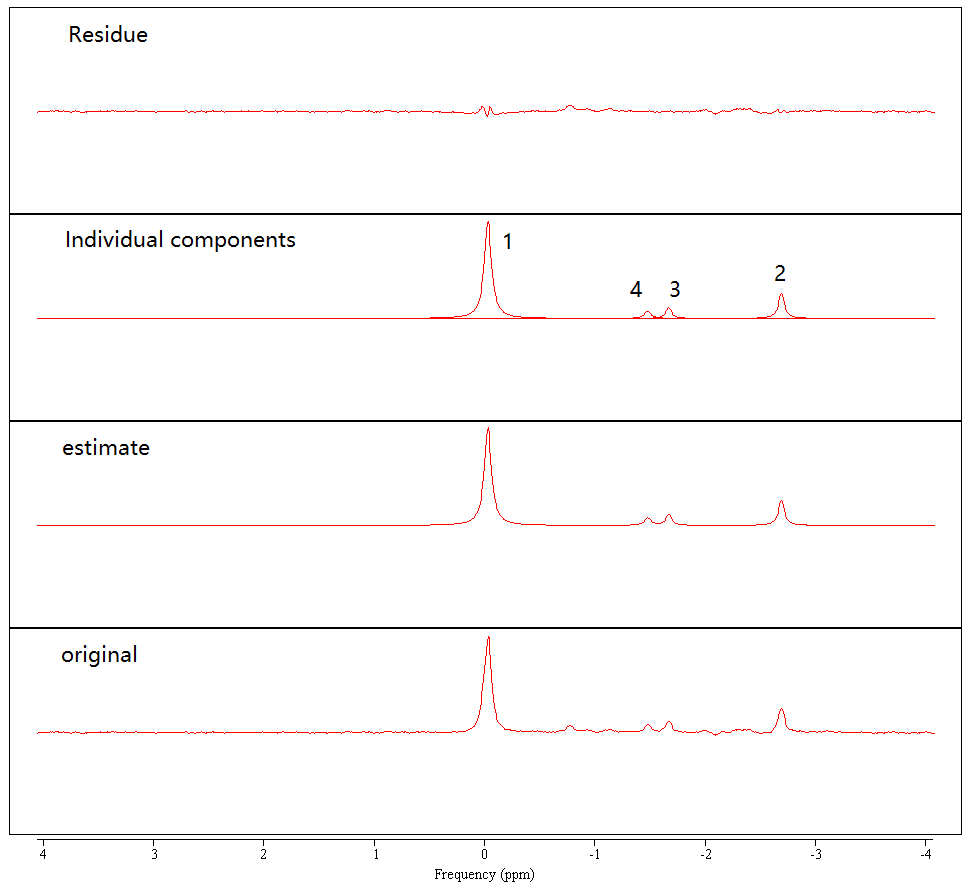


B)

| Temperatures [C] | Ref. linewidth [Hz] | SignalToNoise Ratio |
| --- | --- | --- |
| 38.05 | 7.87667 | 18.08456 |

**Table S2.** Mean and standard deviation of the linewidth and signal-to-noise ratio of four regions. The Water Linew.(Hz), NAA Linew.(Hz), SignalToNoise and S/N were obtained from AMARES fitting and temperature estimation in the JMRUI (See Figure S2).

| Concussion | | | | |
| --- | --- | --- | --- | --- |
|  | Left Occipital | Right Occipital | Left Frontal | Right Frontal |
| Water Linew.(Hz) | 6.76 (0.94) | 6.66 (0.85) | 8.29 (2.31) | 8.07 (1.47) |
| NAA Linew.(Hz) | 6.56 (1.56) | 6.39 (1.49) | 6.03 (1.54) | 6.48 (2.02) |
| SignalToNoise | 10.36 (1.70) | 10.07 (1.03) | 8.80 (1.29) | 8.91 (0.64) |
| S/N | 65.83 (8.64) | 59.88 (10.17) | 50.97 (7.51) | 55.03 (6.25) |

| Control | | | | |
| --- | --- | --- | --- | --- |
|  | Left Occipital | Right Occipital | Left Frontal | Right Frontal |
| Water Linew.(Hz) | 6.83 (0.94) | 6.61 (0.91) | 8.86 (1.80) | 8.25 (1.98) |
| NAA Linew.(Hz) | 6.34 (1.28) | 6.47 (1.59) | 6.89 (2.09) | 8.06 (3.13) |
| SignalToNoise | 10.11 (1.51) | 10.22 (1.01) | 8.61 (0.65) | 8.65 (1.10) |
| S/N | 65.51 (6.65) | 62.88 (10.19) | 54.00 (10.26) | 59.59 (15.13) |
