# Supplementary material for: Selective head cooling in the acute phase of concussive injury: a neuroimaging study
Source: Front Neurol. 2023 Oct 27;14:1272374. doi: 10.3389/fneur.2023.1272374 (PMC10641407; doi:10.3389/fneur.2023.1272374)
Supplement: Supplementary file 3 [file Table_3.DOCX]

**Supplemental File 3**

Schematic of the WElkins sideline cooling system.
