# Supplementary material for: Selective head cooling in the acute phase of concussive injury: a neuroimaging study
Source: Front Neurol. 2023 Oct 27;14:1272374. doi: 10.3389/fneur.2023.1272374 (PMC10641407; doi:10.3389/fneur.2023.1272374)

**Supplemental File 4**

A) the WElkins sideline cooling system on the phantom; B) the volume location of SVS scans (white square) on a structural image; C) the estimated temperature of 36 SVS scans. The dash line is the cooling onset time.


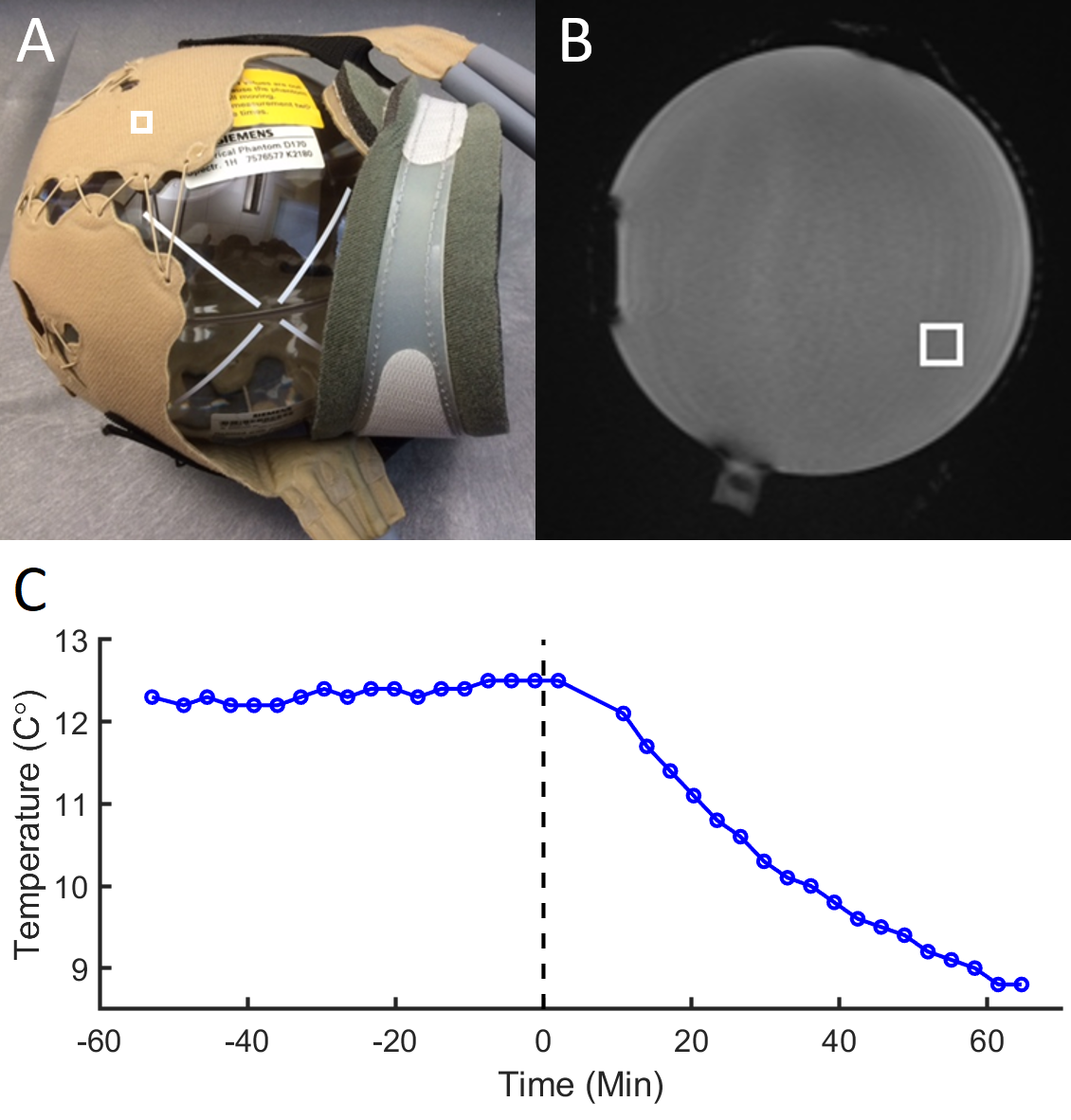

Supplement: Supplementary file 4 [file Table_4.DOCX]
